# A case report of primary multiple hydatid cysts of psoas muscle: An exceptional location

**DOI:** 10.1016/j.idcr.2022.e01637

**Published:** 2022-11-01

**Authors:** Mohamed Ali Chaouch, Bilel Faidi, Maher Hdira, Jihed Rebhi, Nada Feki, Roua Meghri, Aymen Kawach

**Affiliations:** a Department of Visceral and Digestive Surgery, Monastir University Hospital, Monastir University, Tunisia; b Department of General Surgery, Kairouan University Hospital, Sousse University, Tunisia; c Department of General Surgery, Sidi Bouzid Hospital, Tunisia

**Keywords:** Hydatid cyst, Psoas muscle, Hydatidosis, Surgery

## Abstract

The psoas muscle localization is even more exceptional when they are multiple. The clinical and radiological features were often misleading. This case report aimed to highlight the difficulties of radiological diagnosis and the dilemma of choosing the best operative approach. A 27 year-old-men with no past medical history has been consulted for right down abdominal quadrant pain. Physical examination revealed a painless abdominal mass. An abdominal CT Scan concluded with the presence of two multisectional cystic lesions. The first cystic lesion had an exophytic development, and the second cystic lesion was in the posterior and lateral sides of the psoas muscle. The hemaglutinin reaction and the Western blot were positive. A laparotomy was done. The exploration objective is a first psoas muscle hydatid cyst of 5 cm with an exophytic development just behind the vermiform appendix with a second hydatid cyst of 15 cm. The puncturing and aspiration of the cystic fluid bring a clear hydatid fluid. Parasitic sterilization was performed by injecting a scoliosis solution, hypertonic serum, into the cystic lesion. After ten minutes, we resected the two cystic lesions' protruding dome. We have aspirated the fluid and all the daughter hydatid cysts from the two hydatid cysts. The postoperative follow-up was uneventful. The primary hydatid cyst of the psoas muscle often causes a problem of its hydatid nature. Surgery remains the only curative treatment. It avoids the risk of complications such as peritoneal rupture, which can modify the surgical therapeutic strategy.

## Introduction

Muscle hydatid disease is rare, even in endemic countries [1]. The psoas muscle localization, which is part of this entity, is even more exceptional when they are multiple. It represents 0.3% of hydatid cyst locations [2]. The clinical and radiological features were often misleading and confusing [3]. It often poses a problem of seat diagnosis rather than a problem of its hydatid nature despite the great contribution of modern imagery [4]. This case report aimed to highlight the difficulties of radiological diagnosis and the dilemma of choosing the best operative approach.

## Case presentation

**Fig. 1:**
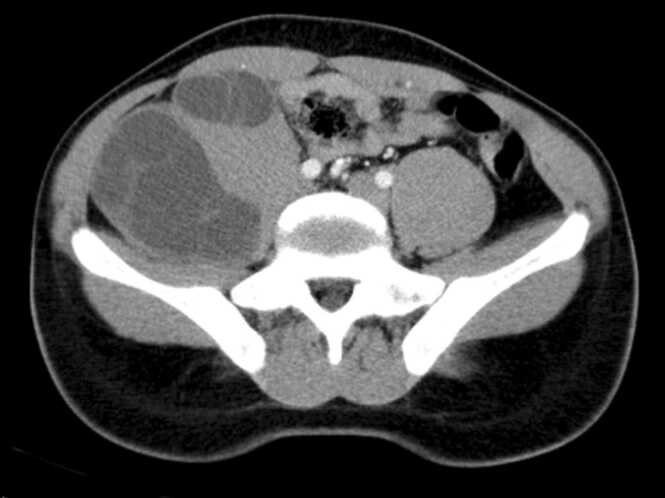
Axial view of abdominal CT scan showing the two psoas muscle hydatid cyst.

A 27 year-old-men with no past medical history has consulted the outpoint clinic for right down abdominal quadrant pain for four months. There was no fever or urinary symptoms. Physical examination revealed a painless abdominal mass measuring five centimetres. Biological data objective a hyper neutrophilia. An abdominal Ct Scan was performed. It concluded with the presence of two multisectional cystic lesions without calcification or infiltration. The first cystic lesion had an exophytic development, and the second cystic lesion was in the posterior and lateral sides of the psoas muscle, measuring 48 and 140 mm, respectively (Fig. 1). The two cysts contain multiple daughter cysts, which suggest hydatid cysts.

**Fig. 2:**
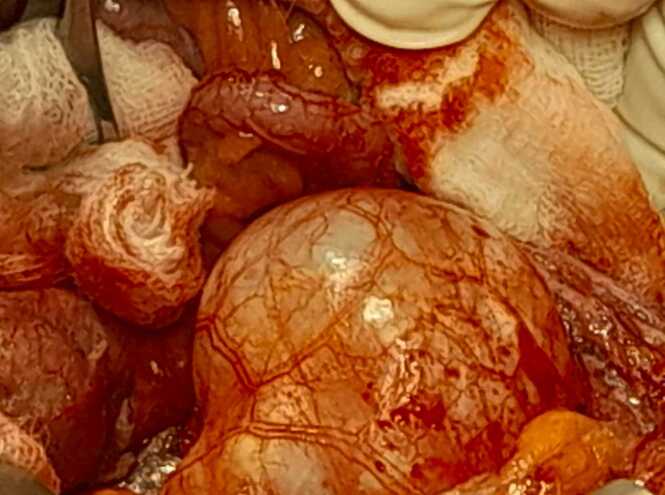
Intraoperative view of the first hydatid cyst with an exophytic development in the peritoneal cavity.

**Fig. 3:**
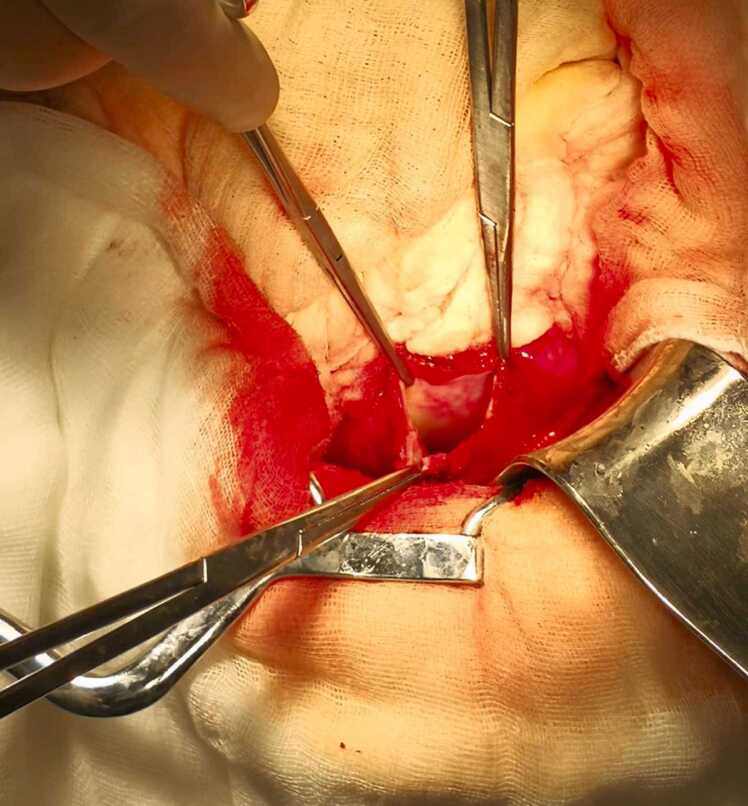
Intraoperative view of the residual hydatid cyst cavity after resection of the protruding dome.

**Fig. 4:**
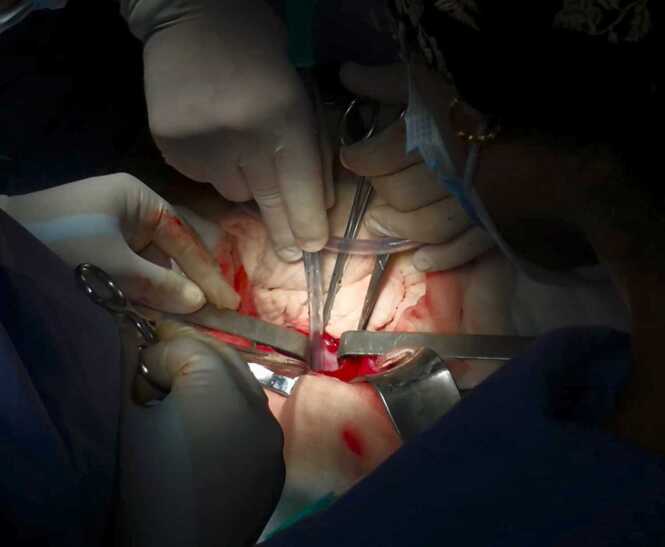
Intraoperative view of the hydatid cyst suction after sterilization of the parasites.

Hydatid cyst serology was performed. The hemaglutinin reaction was positive (1/80), and the Western blot was positive. These radiological and biological data concluded that a psoas muscle hydatid cyst. Surgical management was indicated. A laparotomy was done. There was no liver or peritoneal cystic lesions. The exploration objective is a first psoas muscle hydatid cyst of 5 cm with an exophytic development just behind the vermiform appendix without any adhesion to the adjacent organs (Fig. 2) associated with a second hydatid cyst of 15 cm. We have started the procedure with a lateral to medial right mesocolon dissection to have a better exposition of the two hydatid cysts. We have performed protection of the abdominal cavity by a gauze with a hypertonic serum to avoid peritoneal contamination with the cystic fluid or daughter hydatid cyst dissemination in the peritoneal cavity, causing a postoperative hydatid cyst recurrence. The puncturing and aspiration of the cystic fluid bring a clear hydatid fluid. Parasitic sterilization was performed by injecting a scoliosis solution, hypertonic serum, into the cystic lesion. After 10 min, we resected the two cystic lesions' protruding dome (Fig. 3). We aspirated the fluid and all the daughter hydatid cysts from the two hydatid cysts (Fig. 4). External drainage was used in the residual cavity. The postoperative follow-up was uneventful, and the patient was discharged on the third postoperative day. After three months of follow-up, there is no hydatid cyst recurrence.

## Discussion

Hydatid disease is a public health problem in many Mediterranean countries, the Middle East, New Zealand, India, and South America. Furthermore, it has become a world health problem due to the high flue of an immigrant from these countries. The muscular localization of the hydatid cyst is uncommon [1]. Psoas muscle localization is even rarer and reported in less than 0.5% of cases [2]. The rarity of this localization could be explained by the difficulty of developing the parasitic embryo due to lactic acid production secondary to muscle contractility, which constituted an inadequate condition for embryophore development [3]. Psoas muscle involvement is often primary and occurs via arterial infusion, rarely secondary to an adjacent complicated hydatid cyst [5]. The evolution of the psoas muscle hydatid cyst could be marked by several complications: peritoneal fistula, infection, hydronephrosis and compression of neighboring structures [6], [7], [8], [9]. These complications could have dangerous consequences, like an anaphylactic shock or peritoneal hydatidosis.

Diagnosing hydatid cysts is currently easier due to the imaging and biological features. The abdominal ultrasound showed a cystic lesion sometimes calcified with daughter cysts, with or without membrane detachment, calcified or pseudo-tumoral according to the evolutionary stage of the hydatid disease. Abdominal CT and MRI helped mention the topographic diagnosis of the cyst. They showed a cystic lesion with a proper cystic wall associated with additional specific signs such as the daughter cyst sign [4] or serpent sign [10]. Radiological features could suggest complications: vascular compressions or infections [11] and rule out certain differential diagnoses; then, careful radiological assessment is mandatory. As concern the treatment. It is exclusively surgical. The surgical approach depends on the hydatid cyst location [6], [12]. The extraperitoneal approach is preferred to prevent dissemination into the peritoneal cavity. In our case, a laparotomy was adopted regarding the exophytic development of the first hydatid cyst in the peritoneal cavity, which is difficult to treat using the extraperitoneal approach. The surgical treatment depends on the cyst size. Indeed for small hydatid cysts, generally less than 5 cm radical treatment by a total excision despite sometimes a loss of substance or huge damage to the psoas muscle requiring suture in the majority of cases. For large cysts, conservative treatment by sectioning of the protruding dome appears to be the adequate strategy given the risk of a great damage of the psoas muscle with harmful consequences. The outcome after surgical treatment is often favorable apart from a few complications for ruptured cysts in the such as residual hematic or purulent effusion, and the risk of recurrence.

## Conclusion

The primary hydatid cyst of the psoas muscle often causes a problem of its hydatid nature. Surgery remains the only curative treatment. It avoids the risk of complications such as peritoneal rupture, which can modify the surgical therapeutic strategy.

## Funding

This research received no specific grant from the public, commercial, or not-for-profit sectors.

## Ethical approval

Not applicable.

## Consent

Written informed consent was obtained from the patient for publication of this case report and accompanying images. A copy of the written consent is available for review by the Editor-in-Chief of this journal on request.

## Author contributions

All the authors participate in the treatment of the patients, writing, and approved the manuscript.

## Conflict of interest

No conflict of interest to disclose.
